# Polymer–lipid hybrid nanoparticles as potential lipophilic anticancer drug carriers

**DOI:** 10.1186/s11671-023-03897-3

**Published:** 2023-09-15

**Authors:** Sedef Salel, Banu Iyisan

**Affiliations:** 1 Biofunctional Nanomaterials Design (BiND) Laboratory, Institute of Biomedical Engineering, Bogazici University, 34684 Istanbul, Turkey; 2 Partner Group of Max Planck Institute for Polymer Research Mainz (Germany) at Bogazici University, 34684 Istanbul, Turkey

**Keywords:** Drug delivery systems, Hybrid nanoparticles, Protein/polysaccharide conjugates

## Abstract

Nanocarrier systems are widely used for drug delivery applications, but limitations such as the use of synthetic surfactants, leakage of toxic drugs, and a poor encapsulation capacity remain as challenges. We present a new hybrid nanocarrier system that utilizes natural materials to overcome these limitations and improve the safety and efficacy of drug delivery. The system comprises a biopolymeric shell and a lipid core, encapsulating the lipophilic anticancer drug paclitaxel. Bovine serum albumin and dextran, in various molecular weights, are covalently conjugated via Maillard reaction to form the shell which serves as a stabilizer to maintain nanoparticle integrity. The properties of the system, such as Maillard conjugate concentration, protein/polysaccharide molar ratio, and polysaccharide molecular weight, are optimized to enhance nanoparticle size and stability. The system shows high stability at different pH conditions, high drug loading capacity, and effective in vitro drug release through the trigger of enzymes and passive diffusion. Serine proteases are used to digest the protein portion of the nanoparticle shell to enhance the drug release. This nanocarrier system represents a significant advancement in the field of nanomedicine, offering a safe and effective alternative for the delivery of lipophilic drugs.

**Graphical abstract:**

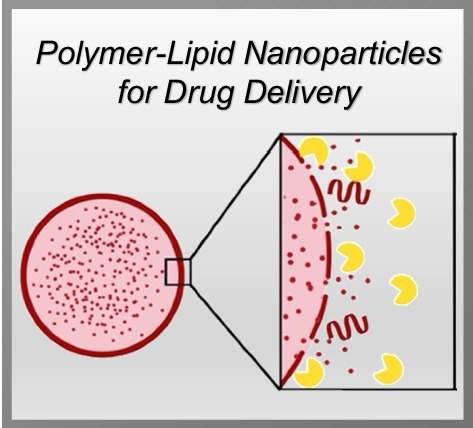

**Supplementary Information:**

The online version contains supplementary material available at 10.1186/s11671-023-03897-3.

## Introduction

Nanocarrier systems have attracted significant attention as an advantageous strategy for drug delivery, as they can overcome the limitations of traditional methods by increasing the bioavailability and stability of drugs, as well as providing controlled and selective delivery capabilities [1]. However, despite progress in the development of these systems, there are still limitations and challenges to be addressed. One major challenge is toxicity associated with synthetic surfactants commonly used in the formulation of nanocarriers [2]. Additionally, controlling drug release is another challenge, as it can greatly impact the efficacy and safety of therapy. Poor control of release kinetics can lead to rapid release of drugs, resulting in poor therapeutic efficacy and increased toxicity [3]. Targeting properties of the nanocarrier systems is also crucial that supports the selectivity of the therapy by differentiating healthy and diseased cells. Besides, the administration of highly lipophilic drugs is also an issue in pharmaceutical field that demands a solution for enhanced therapeutic effects [4, 5]. To improve safety and efficacy of cancer drug delivery, researchers have been searching for promising materials and strategies for the formulation of nanocarrier systems.

Hybrid nanoparticles with a core–shell design are promising for cancer drug delivery due to their ability to utilize the strengths of multiple materials in a single system [6–9]. Among many of these systems, nanoparticles with lipid core and polymeric shell outshines with high drug-carrying capacity and remarkable stability [10]. The lipid core maintains high encapsulation for lipophilic drugs, while the polymeric shell increases the stability of the nanoparticles and protects the drug during blood circulation against external factors [11, 12]. Biopolymers such as polysaccharides and proteins have high molecular weights and could create a physical barrier for the lipid core, providing stability to the nanoparticles in biological environment [13]. Moreover, the biopolymeric shell could be functionalized with targeting agents such as antibodies, peptides, aptamers, or other molecules to improve the targeting ability of the drug delivery system to the specific sites [14]. This can increase the therapeutic efficacy and decrease toxicity of the system for healthy cells. Besides, biopolymers are biocompatible and biodegradable materials, which reduces the risk of toxicity associated with synthetic materials that commonly used in nanocarriers [15].

Bovine serum albumin (BSA) is a commonly used protein in the formulation of nanocarrier systems, due to its several advantages such as amphiphilic structure, biocompatibility, reduction of macrophage uptake, and prolonged half-life in the bloodstream [16]. Furthermore, BSA has been found to accumulate in tumor tissues, leading to a higher drug delivery rate to the targeted cells [17]. In addition, the combination of BSA with natural polysaccharides such as dextran, chitosan, hyaluronic acid, pectin, and alginate enhances the stability of the nanocarriers and helps to avoid undesired drug leakage by changing the diffusion properties of the shell material [18–22]. Among these polysaccharides, dextran takes significant attention because of its biocompatibility, chemical functionality, and potential for decreased immunogenicity [23]. Besides the natural proteins and polysaccharides, natural solid lipids are also drawing attention by being biocompatible, biodegradable, and having efficient encapsulating capacity for lipophilic drugs. For example, mixture of beeswax and carnauba wax was used to form nanoparticles for encapsulating a lipophilic drug, ketoprofen [24], whereas carnauba wax nanoparticles were prepared for the delivery of antioxidants [25]. Although several significant advantages of naked-solid lipid nanoparticles exist, translating them into cancer therapy requires additional coatings for further selective drug delivery. Previously Iyisan et al. reported antibody-functionalized carnauba wax nanoparticles encapsulating a highly hydrophobic drug mimetic for targeting breast cancer cells [26]. Therein, antibodies were adsorbed to the nanoparticle surface by favoring high affinity of the materials to each other. Polymer-shell-solid lipid-core nanocarriers have the potential of extending functionalization opportunities by favoring biopolymers having chemical versatility like BSA and dextran.

Delivery of highly lipophilic anticancer drugs such as paclitaxel is a challenging task in pharmaceutical field [27]. Although paclitaxel is one of the most effective anti-tumor agents acting against variety of cancer types including ovarian cancer, breast cancer, and pancreatic cancer, its poor water solubility leads to challenges during drug administration. The solubilization of paclitaxel in Cremophor EL and ethanol (1:1) is one of the clinically approved ways of paclitaxel commercialization (Taxol) that needs improvement due to the side effects associated with Cremophor EL, a nonionic surfactant to stabilize emulsions. Thus, an alternative carrier system with natural stabilizers and carriers is a need for a safer paclitaxel delivery [28].

In this study, we demonstrate development of hybrid nanocarrier system consisted of a lipid core which includes mixture of beeswax and olive oil and a biopolymeric shell that composed of BSA/dextran Maillard conjugates. While the lipid core provided high encapsulation capacity for a lipophilic drug, the biopolymeric shell served as a stabilizer to the nanocarrier avoiding the use of additional synthetic surfactants. The biopolymeric shell also supported a potential targeting, functionalization, and enzyme-sensitive prospective with its unique material ingredients as albumin and dextran combination. The Maillard conjugates as shell material were characterized using protein assay, sodium dodecyl sulfate–polyacrylamide gel electrophoresis (SDS-PAGE), and Fourier transform-infrared (FT-IR) spectroscopy techniques. Miniemulsion/solvent evaporation method was used to synthesize the solid lipid–polymer hybrid nanoparticles (SLPNs) and they were characterized for their physicochemical and morphological properties. The system was optimized by adjusting different shell properties such as the molecular weight of dextran, BSA/dextran molar ratio, and concentration of the Maillard conjugates. Moreover, size, size distribution, and zeta potential analysis ranging from pH 2 to pH 10 was performed. Finally, paclitaxel, a highly lipophilic anticancer drug, was encapsulated to the solid lipid–polymer hybrid nanoparticles (SLPNs) with high efficiency, and in vitro drug release was evaluated through the trigger of enzymes and passive diffusion.

## Results and discussion

### Formation and characterization of BSA/dextran Maillard conjugates

**Fig. 1:**
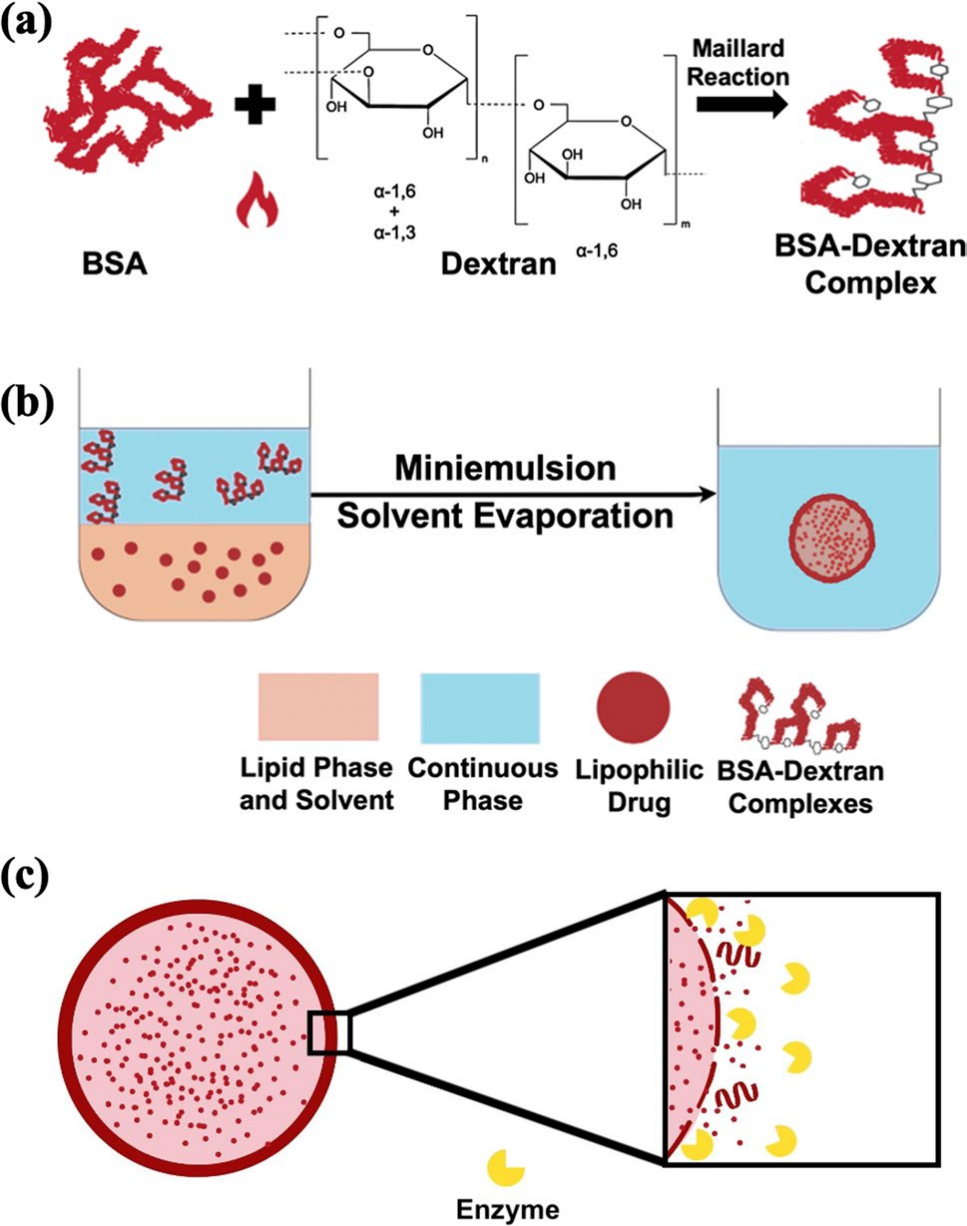
Schematic representation of **a** Maillard complexation reaction between bovine serum albumin (BSA) and dextran. **b** Synthesis of solid lipid–polymer hybrid nanoparticles with lipophilic drug using miniemulsion/solvent evaporation method. **c** Schematic representation of drug release from solid lipid–polymer hybrid nanoparticles via enzymatic triggering

**Fig. 2:**
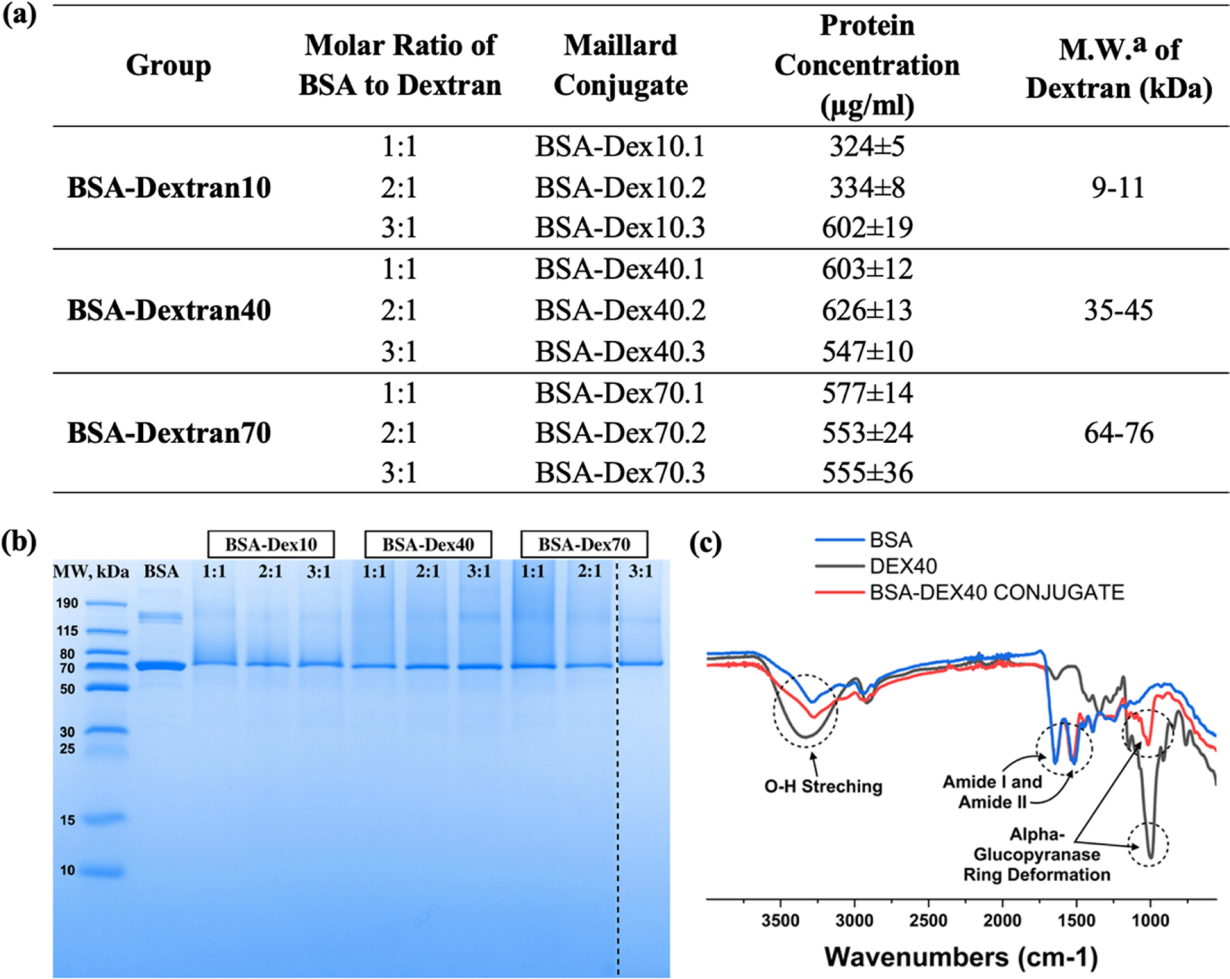
**a** Formulations and protein concentrations of fabricated Maillard conjugates (^a^M.W.: Molecular Weight). **b** SDS-PAGE characterization results of BSA/dextran complexes with different formulations. **c** FT-IR spectrum of BSA (blue), dextran (molecular weight: 40 kDa) (black), Maillard conjugate (BSA-Dex40.2) (red)

BSA and dextran were covalently conjugated via Maillard reaction which is a chemical reaction between the amino groups of proteins and carbonyl groups of reducing carbohydrates [29]. This reaction leads to the covalent conjugation of glucose units with the amine groups, resulting in the formation of N-substituted glycosylamine. Subsequently, the glycosylamine undergoes further transformations, including the formation of Schiff base, which is known to be an unstable compound. In the case of this reaction, the Schiff base that formed is not the final product. It undergoes additional chemical rearrangements known as Amadori rearrangements (Fig. 1). These rearrangements lead to the formation of the final Amadori product. The Amadori product is a stable compound resulting from the rearrangement of the initial Schiff base, and it plays a crucial role in various biological and food-related processes [29, 30]. The steps of chemical changes in the Maillard reaction could be seen in Figure S1. A total of nine different Maillard conjugates were prepared in this study by altering molecular weight (MW_average: 10, 40, 70 kDa) of dextran and molar ratios of BSA to dextran (1:1, 2:1, 3:1) as can be seen in Fig. 2a. Protein assay was performed to find the protein concentrations of each conjugate (Fig. 2a) and the determined concentrations were used to add same amount of protein to each well in SDS-PAGE. SDS-PAGE was performed to support the presence of BSA in the Maillard conjugates. Both BSA and BSA/dextran conjugates showed a dense band around molecular weight of 70 kDa, which corresponded to the molecular weight of native BSA as 66 kDa [31]. Moreover, BSA also showed two other faint bands at high molecular weights which could be because of the clusters of BSA monomers formed due to heating operation during sample preparation for SDS-PAGE (Fig. 2b) [32]. FT-IR spectroscopy was used to examine the binding between BSA and dextran to monitor the BSA/dextran conjugate formation (Fig. 2c). As a result of FT-IR spectroscopy, BSA showed two major characteristic peaks around wavelengths 1520 cm^−1^ and 1650 cm^−1^ which represents the amide 1 and amide 2 stretching vibrations [33]. Dextran spectra demonstrated the characteristic peak around wavelength 3300 cm^−1^ that represents the O–H stretching and the highly sharp peak between wavelengths 800 cm^−1^ and 1100 cm^−1^ that represents the alpha-glucopyranose ring deformation modes [34]. Lastly, the Maillard complex of BSA and different molecular weights of dextran showed the characteristic peaks of both BSA and dextran including the region of amide stretching (1520 cm^−1^, 1650 cm^−1^) that indicates the successful binding of these molecules (Fig. 2c, Figure S2) [35].

### Preparation and characterization of the hybrid nanoparticles

**Fig. 3:**
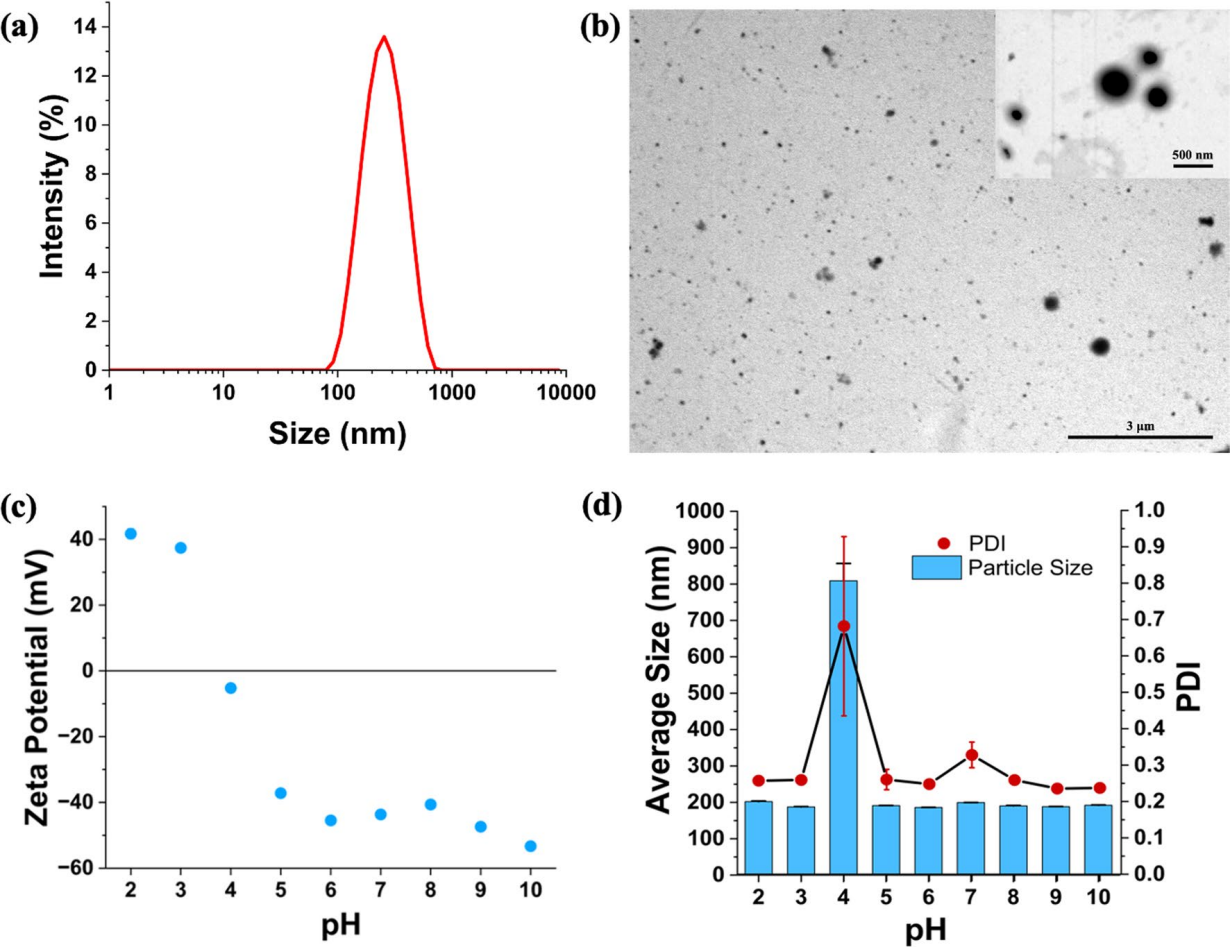
**a** Intensity size distribution of SLPN10.1. **b** Scanning transmission electron microscopy** (**STEM) images of SLPN10.1 show the spherical morphology. **c** pH dependence of zeta potential of SLPN10.1. **d** pH dependence of size and polydispersity index of SLPN10.1. Formulation ingredients of SLPN10.1 are shown in Table 1

Solid lipid–polymer nanoparticles (SLPNs) were synthesized using miniemulsion/solvent evaporation technique (Fig. 1b) [26]. Different shell properties of the SLPNs such as average molecular weight of the dextran (10, 40, 70 kDa) as part of the Maillard conjugates, molar ratio (BSA/dextran, 1:1, 2:1, 3:1), and concentration of the Maillard conjugates (BSA/dextran, 1, 3, 4, 6 mg/mL) were investigated in terms of average size and size distribution values (Table 1). As the molecular weight of dextran increased, the average sizes of the nanoparticles were also slightly increased which could be due to the slight increase in the thickness of the shells. The average sizes were between 215 and 255 nm and polydispersity index values were around 0.25 (Table 1). Variations in the molar ratio of BSA/dextran conjugates did not significantly affect the average sizes of the nanoparticles, although; the smallest nanoparticle was SLPN10.1, and the size distributions of the nanoparticles were similar, and narrowly distributed (Table 1). As the concentration of the Maillard conjugates was increased, the nanoparticle sizes were slightly increased, while their size distributions were becoming narrower. The reason of the size increase could be the slight increment in shell thickness, and the decrease in the polydispersity index could be explained with the denser shell coverage on the surface of the nanoparticles, and consequently, better emulsification and stabilization were performed (Table 1). As a result of all this size and size distribution analysis, SLPN10.1 was chosen as the optimum nanoparticle with the smallest average size (217 nm) and narrow size distribution (PDI: 0.26, Fig. 3a, Table 1) for the further characterization, drug encapsulation, and drug release studies. Furthermore, it is valuable to mention that the obtained nanoparticles in this study had similar or smaller size and size distribution values than other reported solid lipid nanoparticle systems formed with synthetic surfactants or emulsifiers [36–38]. This supported the stabilization ability of BSA/dextran conjugates in miniemulsions that provided intact nanoparticle synthesis without using synthetic surfactants. Scanning transmission electron microscopy (STEM) was used to image the morphological structure of SLPN10.1. Spherical shapes of the nanoparticles were supported in STEM images (Fig. 3b). STEM images were analyzed through ImageJ program and the histogram for the diameters of the nanoparticles was produced (Figure S5). The average size of the nanoparticles according to histogram analysis was 213 nm. Thus, the sizes of the nanoparticles that were observed in STEM were compatible with dynamic light scattering results.

**Table 1:** Particle size and polydispersity index dependency of the nanoparticles to changes in molecular weight of dextran, molar ratio (BSA/dextran), and concentration of Maillard complexes

Shell parameters	Nanoparticle*	Shell: BSA/dextran Maillard conjugate	Average size (nm)	Polydispersity index
Molecular weight of dextran	SLPN10.1	BSA-Dex10.1	217.0 ± 2.2	0.26 ± 0.009
	SLPN40.1	BSA-Dex40.1	240.8 ± 1.5	0.35 ± 0.006
	SLPN70.1	BSA-Dex70.1	253.3 ± 3.8	0.24 ± 0.012
Molar ratio (BSA/dextran)	SLPN10.1	BSA-Dex10.1	217.0 ± 2.2	0.26 ± 0.009
	SLPN10.2	BSA-Dex10.2	256.6 ± 2.9	0.28 ± 0.004
	SLPN10.3	BSA-Dex10.3	235.7 ± 2.6	0.24 ± 0.010
Concentration of Maillard complexes	SLPN10.1 (Shell: 1 mg/mL)	BSA-Dex10.1	217.0 ± 2.2	0.26 ± 0.009
	SLPN10.1 (Shell: 3 mg/mL)	BSA-Dex10.1	220.0 ± 1.6	0.24 ± 0.003
	SLPN10.1 (Shell: 4 mg/mL)	BSA-Dex10.1	222.7 ± 5.7	0.22 ± 0.003
	SLPN10.1 (Shell: 6 mg/mL)	BSA-Dex10.1	227.3 ± 2.0	0.17 ± 0.028

*Core content: Beeswax (40%) and Olive Oil (60%)

### pH-dependent zeta potential and size analysis of SLPN10.1

To investigate size, size distribution, and zeta potential of SLPN10.1 in different pH values, the nanoparticle was titrated against NaOH (0.1 M) and HCl (0.1 M) solutions, and dynamic and electrophoretic light scattering measurements of the nanoparticle in each pH between 2 to 10 were performed. According to zeta potential analysis of SLPN10.1, in acidic conditions below pH 4, the nanoparticle had strongly positive zeta potential values, while above pH 4; it had strongly negative zeta potential values (Fig. 3c). Another outcome here was that the isoelectric point of the nanoparticle was close to pH 4, which is rational since isoelectric point of BSA is 4.7 [39], and BSA is the predominant material in the shell of the hybrid nanoparticle. BSA amount is much higher than dextran in SLPN10.1 since molecular weight of BSA (66 kDa) is much higher than molecular weight of dextran10 (9–11 kDa). As zeta potential was higher than 30 (positive or negative) in each pH condition except pH 4, the nanoparticle was electrostatically stable in these pH values [40]. Moreover, the nanoparticle size and size distribution values were very high at pH 4 which could be due to the precipitation of the nanoparticles since there is no charge in the surface and as a result there is not enough repulsion forces between nanoparticles in the isoelectric point. In other pH values, average sizes were between 185 and 205 nm, and size distributions were between 0.23 and 0.30 (Fig. 3d).

It should be also noted that the zeta potential values of the SLNP10.1 at pH 8 were monitored up to 4 months to understand the colloidal stability of the system. The zeta potential values were almost stable within this time ranging between − 39 and − 43 mV (Table S1). Besides, the size variation of the nanoparticles was almost negligible and the SLPN10.1 nanoparticles kept their integrity without any macroscopic phase separation over 4 months. Furthermore, to compare the performance of our surfactant-free miniemulsion templating approach, a control experiment was conducted. This involved fabricating nanoparticles using a 1% (v/v) concentration of Tween 20 surfactant in the continuous phase (SLPN_TW20, Figure S6). The evaluation of this nanoparticle includes size measurements, size distribution analysis, zeta potential determinations, and detailed microscopic analysis through scanning transmission electron microscopy (STEM) imaging (Figure S6). The control nanoparticles have a smaller average size (SLPN_TW20: 143 nm, PDI:0.25) than the nanoparticles formulated using our natural biopolymers (SLPN10.1: 217 nm, PDI:0.26) as expected. This difference in size is because the latter one possesses an additional protective biopolymer shell, which contributes to the overall size. On the other side, the resultant nanoparticles from both groups exhibited similar colloidal stability (Table S1), maintained low polydispersity index (PDI) values, and demonstrated identical morphological features. This close resemblance underlines the indispensable role that BSA/dextran conjugates play as highly efficient stabilizers, effectively protecting the physicochemical integrity of the solid lipid core within the hybrid nanoparticles, all without the need for synthetic surfactants.

### Paclitaxel encapsulation and in vitro drug release studies

Before the drug release experiments, size, size distributions, and zeta potentials of the nanoparticles were determined at pH 6.5 and at pH 7.4 in phosphate buffers to mimic early endosomal and physiological pH conditions, respectively.

**Fig. 4:**
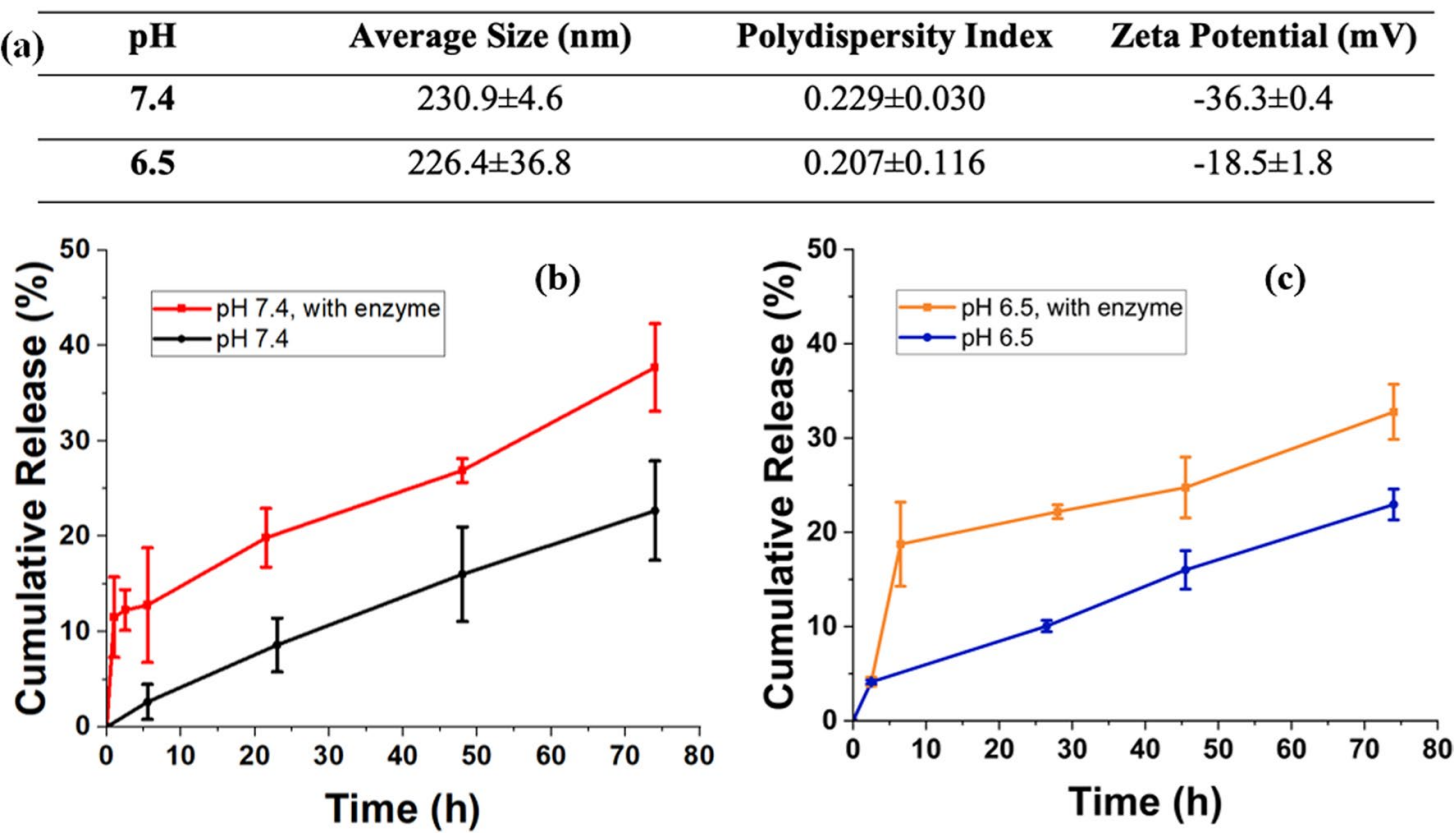
**a** Size, PDI, and zeta potential values of SLPN10.1 in phosphate buffers (pH 7.4 and 6.5). **b** Cumulative release of paclitaxel through passive diffusion and enzymatic triggering in pH 7.4. **c** Cumulative release of paclitaxel through passive diffusion and enzymatic triggering in pH 6.5. (protein/serine protease ratio: ½)

The results showed that the average size and polydispersity index of the nanoparticles were similar, and highly monodisperse nanoparticles were obtained. The sizes were around 230 nm for both pH values (Average sizes: 230.9 ± 4.6 (pH 7.4), 226.4 ± 36.8 (pH 6.5)) and there was no significant size variation (PDI values: 0.229 ± 0.030 (pH 7.4), 0.207 ± 0.116 (pH 6.5)) in buffer solutions in comparison to the nanoparticles dispersed in ultrapure water. (Fig. 4a, Table 1). Zeta potential of the nanoparticles at pH 6.5 and 7.4 was -18.5 and -36.3, respectively (Fig. 4a). The results indicated that the nanoparticles were stable at both physiological pH (pH 7.4) and early endosomal pH (pH 6.5) mimicking conditions and thus in vitro drug release studies were conducted as a next step.

Paclitaxel, an anticancer drug used to treat breast, ovarian, pancreatic, and lung cancer [41], was selected for this study to be encapsulated into the hybrid nanoparticle system. To calculate encapsulation efficiencies, excessive centrifugation steps were applied to the nanoparticles to ensure that nonencapsulated drugs were removed effectively. Then, the nonencapsulated paclitaxel was collected in the supernatant which is followed by calculating paclitaxel concentration of supernatants from the absorbances in 260 nm using calibration curves for pH 6.5 and pH 7.4 (see Figure S4). Finally, the efficiencies were calculated using the encapsulation efficiency equation (Eq. 1). Encapsulation efficiency of the nanoparticle was 85 ± 0.5% for pH 7.4, and 65 ± 0.8% for pH 6.5. Similar results were observed previously in which paclitaxel encapsulation efficiency of PX G78 (composed of glyceryl tridodecanoate (GT) and polyoxyethylene 20-stearyl ether (Brij 78)) nanoparticles were 85%, whereas the drug release was around 25% within 80 h at pH 7.4 [42]. The unique design of our nanoparticles, which involves the combination of natural materials without the use of a synthetic surfactant, sets them apart from other drug delivery systems that utilize different materials. Furthermore, our study provides valuable insights into the effects of the serine protease enzyme on drug release, emphasizing the importance of enzymatic triggering for controlled release, and examines the encapsulation efficiency and drug release results both in physiological (pH 7.4) and early endosomal conditions (pH 6.5). The higher encapsulation efficiency at pH 7.4 may be attributed to the higher interactions of the drug in this condition as supported by highly negative charge obtained from zeta potential analysis. Cumulative drug release profiles of SLPN10.1 were evaluated in pH 7.4 and pH 6.5 phosphate buffers. For these two pH values, drug release with passive diffusion and enzymatic triggering were observed. For the enzymatic triggering method, serine protease triggered release is a promising approach in cancer therapy. These enzymes are overexpressed in cancer cells and contribute to tumor growth, invasion, and metastasis. By designing drug delivery systems that respond to the activity of serine proteases, targeted release of anticancer drugs within the tumor microenvironment becomes possible [43]. The development of innovative serine protease triggered release systems is an active area of research with the aim of advancing clinical applications in cancer treatment [44, 45]. In this study, serine protease enzyme was used to digest the protein portion of the nanoparticle shell to enhance the drug release as protein/enzyme ratio was ½ (w/w). For both pH values, drug release with enzymatic triggering was significantly higher than passive diffusion that is promising to minimize the side effects of the drugs in healthy cells in future studies. At pH 7.4, the cumulative release result of the nanoparticle at the end of 74 h was 22.7 ± 5.2 with passive diffusion, and 37.7 ± 4.6 with enzymatic triggering (Fig. 4b). At pH 6.5, the cumulative release result of the nanoparticle at the end of 74 h was 22.9 ± 1.6 with passive diffusion and 32.7 ± 2.9 with enzymatic triggering (Fig. 4c). These results show that serine protease enzyme effectively cleaves the peptide bonds of BSA and breaks down the protein into smaller amino acids, so that paclitaxel could leak more easily to the environment. The clear contribution of enzymes in accelerating and controlling the drug release was also confirmed through statistical analysis (see SI).

## Conclusion

In this study, a hybrid core–shell type nanoparticle system was fabricated with a nanostructured lipid core and a biopolymeric shell which consist of BSA/dextran Maillard conjugates. It has been seen that BSA/dextran Maillard conjugates served as stabilizers to the nanoparticles and thus; stable, small sized, highly monodisperse nanoparticles obtained without the use of synthetic surfactants. Size and size distribution analysis of nanoparticles having different shell properties such as molecular weight of dextran (10, 40, 70 kDa), molar ratio (BSA/dextran: 1:1, 2:1, 3:1), and concentration of the Maillard conjugates (BSA/dextran: 1, 3, 4, 6 mg/mL) were performed to obtain the optimum nanoparticle in terms of size and polydispersity index. SLPN10.1 (Nanoparticle shell: BSA/dextran10 (dextran M.W.: 10 kDa), Molar ratio = 1:1) was chosen as the optimum nanoparticle. It showed spherical morphology, small size (217 nm) that can induce enhanced permeability and retention (EPR) effect to indirectly target tumor sites, and narrow size distribution (PDI: 0.26). pH-Dependent zeta potential analysis showed that the nanoparticle was highly stable in each pH between 2 and 10, except pH 4 which is the isoelectric point of the nanoparticle. Paclitaxel, a highly lipophilic anticancer drug, was encapsulated to the nanoparticle with high encapsulation efficiencies (85 ± 0.5% (pH 7.4), 65 ± 0.8% (pH 6.5)), and drug release performed through the mechanism of passive diffusion and enzymatic triggering. Higher drug release was observed with enzymatic triggering approach than passive diffusion in both pH 7.4 (physiological condition) and pH 6.5 (tumor microenvironment condition), so we can conclude that controlling the drug delivery with the aid of enzymes has a high potential and can be further extended. Overall, this study presents a novel hybrid core–shell nanoparticle system comprising natural lipids and polymers synthesized without the need for synthetic surfactants for the delivery of lipophilic drugs. The BSA/dextran Maillard conjugates used in the shell provide stability and control over nanoparticle properties, resulting in optimized characteristics such as small size, pH-stable behavior, and efficient drug encapsulation. The system's ability to achieve controlled drug release through enzymatic triggering shows its potential in improving therapeutic outcomes. Looking ahead, this hybrid nanocarrier system holds promise not only for safer and more effective drug delivery in cancer treatment but also as a platform for advancing drug delivery strategies across diverse medical applications.

## Experimental section

### Materials

Bovine serum albumin (BSA) (purity: > 98%), olive oil (highly refined, low acidity), beeswax, and hydrochloric acid (HCl) were purchased from Sigma-Aldrich Company. Paclitaxel, dextran 10 (MW:9000 to 11,000), dextran 40 (MW: 35,000 to 45,000), and dextran 70 (MW: 64,000 to 76,000) were purchased from Carbosynth. Ethyl acetate (ACS grade), potassium bromide (KBr), potassium chloride (KCl), monobasic sodium phosphate (NaH_2_PO_4_·H_2_O), dibasic sodium phosphate (Na_2_HPO_4_·7H_2_O), and sodium hydroxide (NaOH) were provided from Acros Organics. Pierce 660 nm protein assay reagent and SDS-PAGE materials; 4X Bolt LDS sample buffer, 10X Bolt reducing agent, 20X Bolt MES SDS running buffer, Bolt Bis–Tris running gels (10%, 10-well), and Invitrogen SimplyBlue safestain (Coomassie Brilliant Blue) were purchased from ThermoFisher Scientific.

### Preparation of BSA/dextran conjugates

BSA/dextran conjugates were prepared via Maillard reaction that is a complex reaction occurring between proteins and polysaccharides with the assistance of heat (Fig. 1a) [46]. Amino groups of BSA and glucose units of dextran were covalently conjugated with this reaction. The conjugates were prepared according to the method described by Kim and coworkers (2003) with slight modification [47]. Firstly, 10 mL of BSA/dextran mixtures in molar ratios seen in Fig. 2a was prepared in ultrapure water with 1% (w/v). The pH of the mixtures was adjusted to 8 using 0.1 M Sodium Hydroxide (NaOH) and they were stirred for 16 h. Then, the mixtures were lyophilized and were put to incubator inside a desiccator containing saturated potassium bromide (KBr) solution to maintain 79% relative humidity for 48 h at 60 °C. The resulting products were defined as Maillard conjugates and stored at + 4 °C for further characterization and usage.

### Protein assay

Thermo Scientific Pierce 660 nm Protein Assay was selected for the characterization of BSA/dextran Maillard complexes because of its easiness, quickness, and being more linear than Bradford assays. BSA was used as the standard for the calibration curve (Figure S3). To perform the assay, firstly 1 mg/mL sample solutions were prepared using ultrapure water. Then, 10 μL of each replicate of standard, unknown sample, and the appropriate blank sample (ultrapure water) was added into a microplate well (96 well-plates). 150 μL assay reagent was added to each well. The plate was shaken in medium speed for one minute and then the absorbances were measured. Ultrapure water was used as blank, and the protein concentrations of each sample were calculated according to the calibration curve. Each absorbance measurement was performed triplicate.

### Sodium dodecyl sulfate–polyacrylamide gel electrophoresis (SDS-PAGE)

SDS-PAGE was conducted for confirmation of Maillard complexation between BSA and dextran in Maillard conjugates. Each of the conjugates was dissolved in ultrapure water at a concentration of 1 mg/mL. Conjugate samples and 0.5 mg/mL BSA solution were mixed with 2.5 μL of Bolt LDS Sample Buffer, 1 μL of Bolt Sample Reducing Agent, and ultrapure water (if necessary), and their total volume was 10 μL. BSA concentrations in each conjugate sample were known from protein assay results and they used to calculate the sample amounts to be used for obtaining 2.5 μg BSA in each well of the gel. The sample solutions were heated at 70 °C for 10 min prior to gel loading. After addition of Bolt MES SDS Running Buffer into tank, BSA and each sample were loaded into wells of a 10% Bis–Tris protein gel. Running was performed at constant 40 mA for 90 min using PageRule Plus Prestained Protein Ladder as a ladder. SimplyBlue SafeStain was used to stain the gel by overnight stirring on a gentle shaker and next day the gel was rinsed with 200 mL ultrapure water for 1 h, twice. Lastly, the gels were scanned and imaged using a scanner (Bio-Rad Gel Doc XR + Gel Documentation System).

### Fourier transform-infrared spectroscopy (FT-IR)

FT-IR spectroscopy was performed to analyze chemical features and structures of BSA, dextran and Maillard conjugates using a Nicolet 380 FT-IR spectrometer (Thermo Scientific). The spectrums were collected between the wavenumbers 400 cm^−1^ and 4000 cm^−1^ and analyzed using OMNIC software.

### Nanoparticle synthesis

Solid lipid–polymer hybrid nanoparticles (SLPNs) were synthesized by direct miniemulsion-solvent evaporation technique (Fig. 1b). In this technique, nanoparticles were prepared by dispersion of the oil phase including dissolved lipids in a solvent within the aqueous phase which contains preformed biopolymeric conjugates, and followed by the evaporation of the solvent [26]. In this study, olive oil (60 mg), beeswax (40 mg), and ethyl acetate (2 mL) were used as dispersed phase in oil-in-water emulsion, while BSA/dextran conjugates (1 mg/mL) were used as a continuous phase (10 mL). The two phases were stirred at 1000 rpm for 2 min, and further ultrasonication was performed for 4 min under ice bath at 90% amplitude (15 s on, 5 s off) with Branson 550 Sonifier. The ethyl acetate was evaporated at room temperature along with stirring at 150 rpm for 16 h. Specifications of these nanoparticles could be seen in Table1.

### Dynamic light scattering (DLS)

Dynamic light scattering (DLS) measurements were taken with Zetasizer Nano ZS (Malvern Instruments) with a fixed scattering angle of 173°. Each nanoparticle was diluted before measurements. The size of the nanoparticles is measured as intensity-average diameter (z_average_) values assuming the refractive index of the nanoparticles are 1.47. Size and size distributions of the nanoparticles were investigated with different shell parameters of the hybrid nanoparticles such as molecular weight of dextran, molar ratio of BSA/dextran, and concentration of Maillard conjugates. Zeta potential of the nanoparticles were determined by titration against HCl (0.1 mol/L) and NaOH (0.1 mol/l) solutions. During this titration, the size and size distributions of the nanoparticles were also measured to observe the stability of the nanoparticles.

### Scanning transmission electron microscopy (STEM)

Scanning transmission electron microscopy (STEM) measurements were performed to observe the morphology of SLPN10.1 with Quattro S (Thermo Scientific). For the measurements, 3 μL sample was dried on a 300-grid copper mesh for 60 min prior to measurements.

### Paclitaxel encapsulation

Based on the procedure in the previous study that doxorubicin (an anticancer drug) was encapsulated in nanoparticles [48], and considering drug amounts of other studies that consist of paclitaxel-encapsulated lipid nanoparticles [42], drug loading in this study was performed with 0.1 mg/mL paclitaxel. Paclitaxel (1 mg) was mixed with the lipid phase and solvent before the ultrasonication of the two phases of the miniemulsion. The rest of the synthesis was identical as explained for the nanoparticle synthesis without drug. Formed nanoparticles were purified from nonencapsulated paclitaxel via centrifugation at 20,000 rpm for 1 h at room temperature. Centrifugation was applied 2 times to ensure the purity of the miniemulsion. To calculate the encapsulation efficiency of the nanoparticles, drugs in the supernatant were collected and absorbance values at maximum point of the corresponding drug were measured for both supernatant and the nanoparticles after the centrifugation step by using ultraviolet and visible light (UV–Vis) spectroscopy (NanoDrop2000c, ThermoFisher Scientific). The drug encapsulation efficiency of the hybrid nanoparticles was calculated with the following equation:

1 $${\text{Encapsulation }}\;{\text{Efficiency }}\left( {\text{\% }} \right) = { }\frac{{{\text{Initial}}\;{\text{ Drug}}\;{\text{ Amount}} - {\text{Free }}\;{\text{Drug}}\;{\text{ Amount}}}}{{{\text{Initial }}\;{\text{Drug }}\;{\text{Amount}}}} \times 100{\text{\% }}$$

### In vitro paclitaxel release

In vitro paclitaxel release was performed with two different approaches: passive diffusion and enzymatic triggering (Fig. 1c). For drug release with passive diffusion, paclitaxel-encapsulated nanoparticles (3 mL) at pH 6.5 which is the pH around tumor microenvironment [49], and pH 7.4 which is normal physiological pH of the blood, were placed in dialysis membranes (molecular weight cutoff (MWCO): 6000–8000). For drug release with enzymatic triggering, paclitaxel-encapsulated nanoparticles at pH 6.5 and 7.4 (3 mL) were incubated with serine protease enzyme (protein/enzyme ratio:1/2) and placed in dialysis membranes (MWCO: 6000–8000). All of them were then placed in 2 L buffer-containing beaker at body temperature (37 °C) and stirring (200 rpm) conditions. The release media was prepared as phosphate buffers (0.01 M) at either pH 7.4 or pH 6.5 including 0.1% Tween80 (v/v) [42]. For measuring the drug release, 10 μL of sample was taken at predefined time intervals for UV–Vis analysis. The amount of remaining paclitaxel in nanoparticles at each sampling point was then calculated from the calibration curves at pH 6.5 and pH 7.4 (Figure S4). The cumulative paclitaxel release at each time interval was obtained from the equation below:

2 $${\text{Cumulative}}\;{\text{ Release}}\left( {\text{\% }} \right) = \frac{{{\text{Initial }}\;{\text{Drug }}\;{\text{Amount}} - {\text{Remaining }}\;{\text{Drug }}\left( {{\text{at }}\;{\text{each }}\;{\text{sampling}}\;{\text{ point}}} \right)}}{{{\text{Initial }}\;{\text{Drug }}\;{\text{Amount}}}} \times 100{\text{\% }}$$

### Supplementary Information

**Additional file 1.**

## Data Availability

The authors declare that the data supporting the findings of this study are available within the paper and its Supporting Information files.
