# Supplementary material for: Polymer–lipid hybrid nanoparticles as potential lipophilic anticancer drug carriers
Source: Discov Nano. 2023 Sep 15;18(1):114. doi: 10.1186/s11671-023-03897-3 (PMC10504175; doi:10.1186/s11671-023-03897-3)
Supplement: Supplementary file 1 — Additional file 1. [file 11671_2023_3897_MOESM1_ESM.pdf]

Supporting Information

Polymer-Lipid Hybrid Nanoparticles as Potential Lipophilic Anticancer Drug Carriers

Sedef Salel[1], *Banu Iyisan*[1,2]*

[1]Biofunctional Nanomaterials Design (BiND) Laboratory, Institute of Biomedical Engineering, Bogazici University, 34684, Istanbul, Turkey

[2]Partner Group of Max Planck Institute for Polymer Research Mainz (Germany) at Bogazici University, 34684, Istanbul, Turkey

*Corresponding author E-mail: banu.iyisan@boun.edu.tr

Table of Contents for Supporting Information:

1. Supporting Figures

Figure S1. Scheme of Maillard Reaction between bovine serum albumin (BSA) and dextran.

Figure S2. FT-IR spectrum of (a) BSA, Dextran (molecular weight: 10 kDa), BSA-Dex10.2 and (b) BSA, Dextran (molecular weight: 70 kDa), BSA-Dex70.2.

Figure S3. Calibration Curve of BSA determined from absorbance values at λ = 655 nm to calculate protein concentrations of each Maillard complex.

Figure S4. Calibration Curve of Paclitaxel determined from absorbance values at λ = 260 nm at (a) pH 6.5, (b) pH 7.4.

Figure S5. Size Distribution Histogram of the Nanoparticles obtained from Scanning Transmission Electron Microscopy Images

Figure S6. (a) Size, Polydispersity index (PDI), and zeta potential values of SLPN_TW20 (control nanoparticles synthesized using %1 v/v Tween 20). (b) Scanning Transmission Electron Microscopy (STEM) images of SLPN_TW20 show the spherical morphology.

Figure S7. FT-IR spectrum of nanoparticles: SLPN10.1

Table S1. Zeta potential values of SLPN10.1 and SLPN_TW20 (control nanoparticles) over the storage time of four months. Formulation ingredients of SLNP10.1 is in Table 1.

2. Statistical Analysis

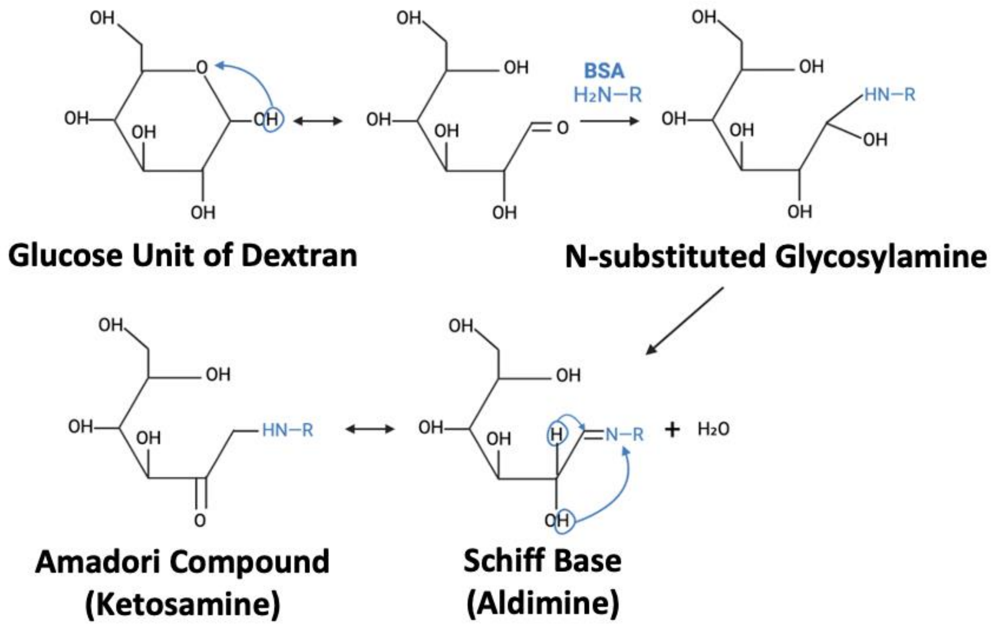

Figure S1. Scheme of Maillard Reaction between bovine serum albumin (BSA) and dextran Adapted from [1].

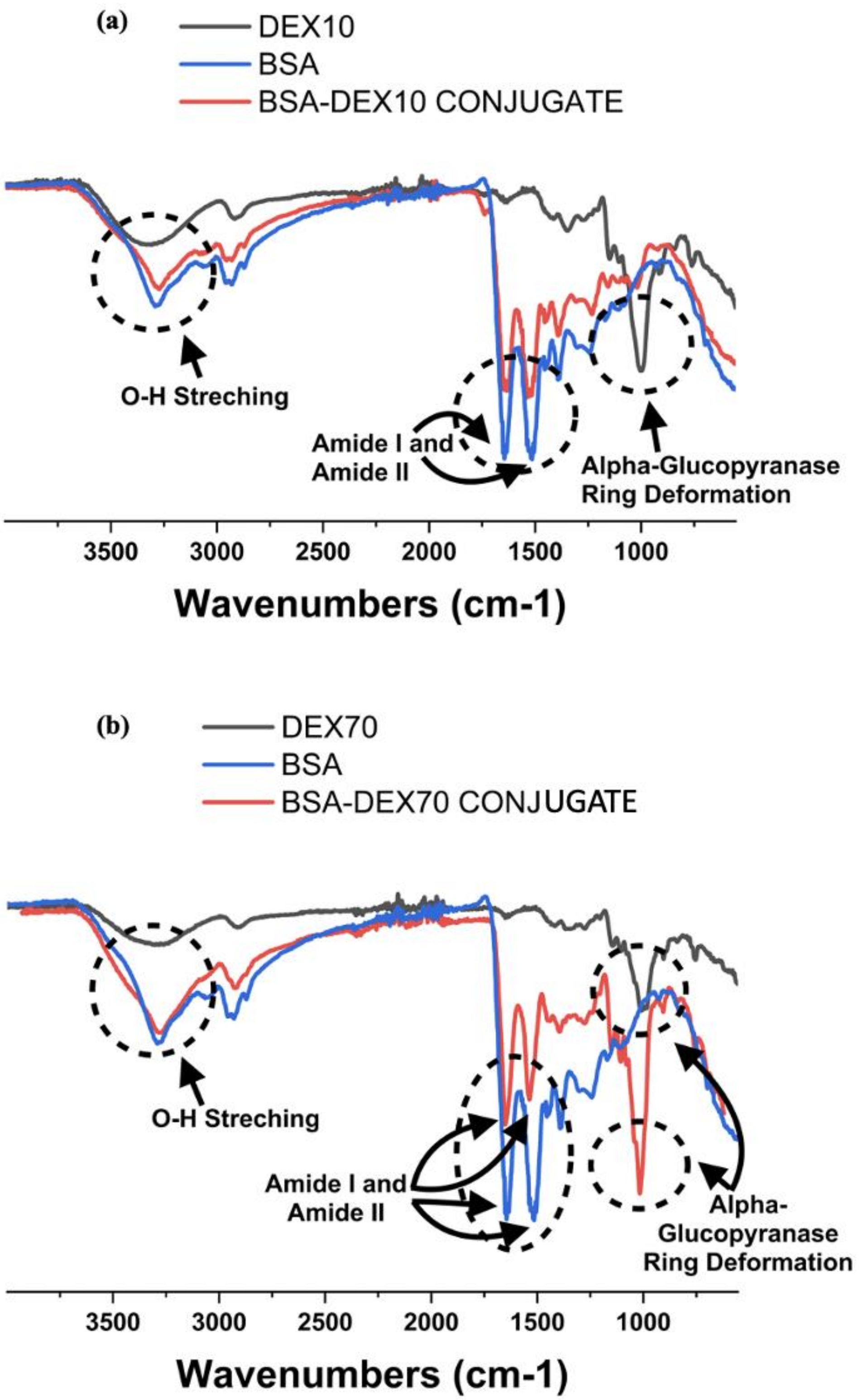

Figure S2. FT-IR spectrum of (a) BSA (blue), Dextran (molecular weight: 10 kDa) (black), BSA-Dex10.2 (red) and (b) BSA (blue), Dextran (molecular weight: 70 kDa) (black), BSA-Dex70.2 (red).

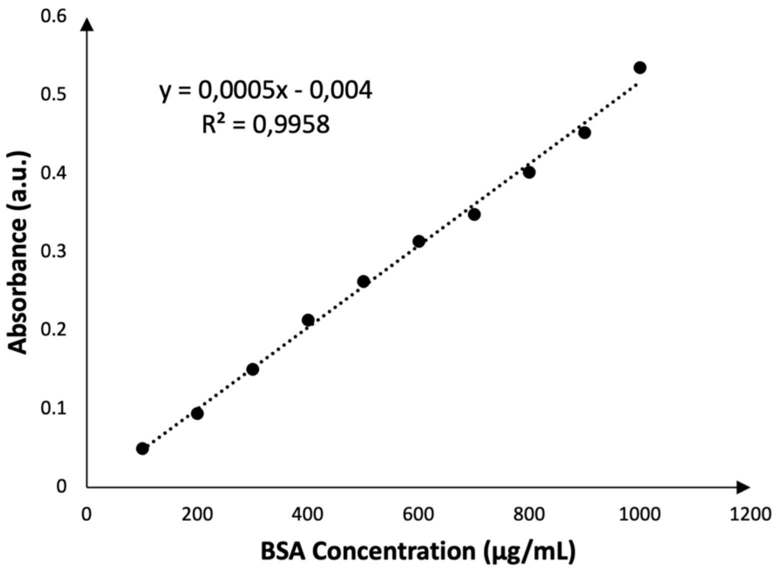

Figure S3. Calibration Curve of BSA determined from absorbance values at λ = 655 nm to calculate protein concentrations of each Maillard complex.

(a)

(b)

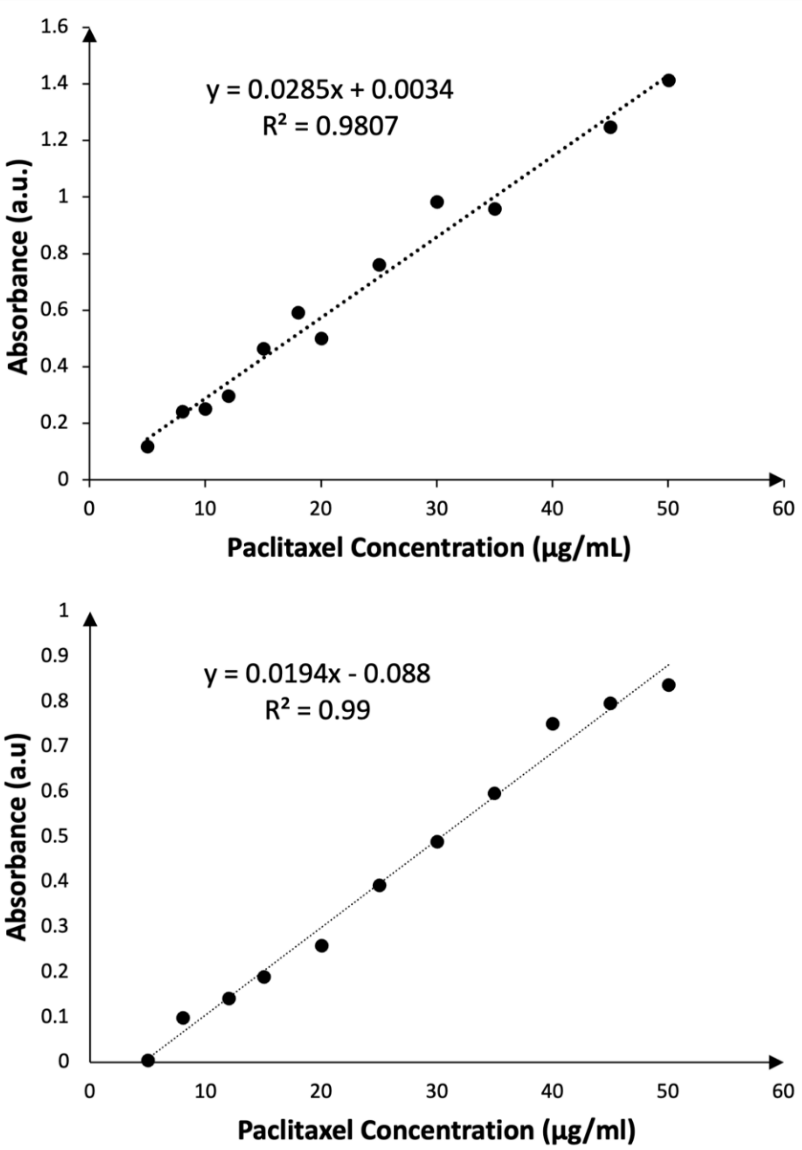

Figure S4. Calibration Curve of Paclitaxel determined from absorbance values at λ = 260 nm at (a) pH 6.5, (b) pH 7.4.

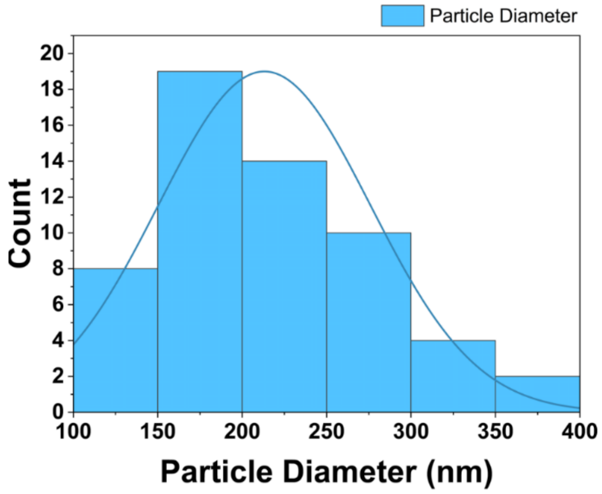

Figure S5. Size Distribution Histogram of the Nanoparticles obtained from Scanning Transmission Electron Microscopy Images

(a)

Average Size (nm)	Polydispersity Index	Zeta Potential (mV)
143.2 ± 0.8	0.25 ± 0.03	-29..9 ± 2.1

(b)

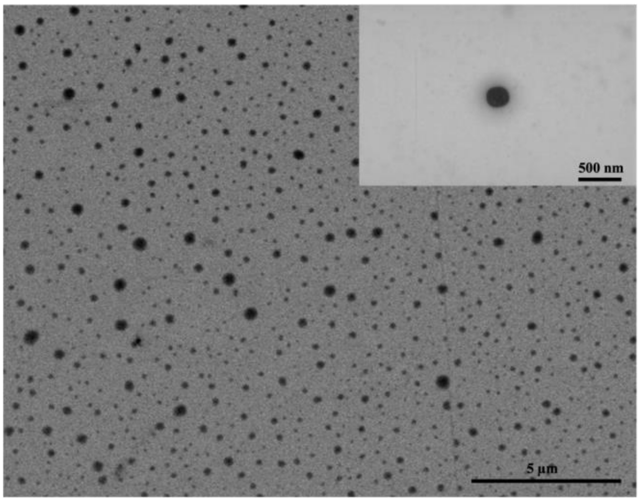

Figure S6. (a) Size, Polydispersity index (PDI), and zeta potential values of SLPN_TW20 (control nanoparticles synthesized using %1 v/v Tween 20). (b) Scanning Transmission Electron Microscopy (STEM) images of SLPN_TW20 show the spherical morphology

Table S1. Zeta potential values of SLPN10.1 and SLPN_TW20 (control nanoparticles) over the storage time of four months. Formulation ingredients of SLNP10.1 is in Table 1.

Stability Check	SLPN10.1		SLPN_TW20	
	**t = 0**	**t = 4 months**	**t = 0**	**t = 4 months**
**Zeta Potential (mV)**	-40.7 ± 0.1	-42.2 ± 0.4	-29.9 ± 2.1	-27.9 ± 0.5

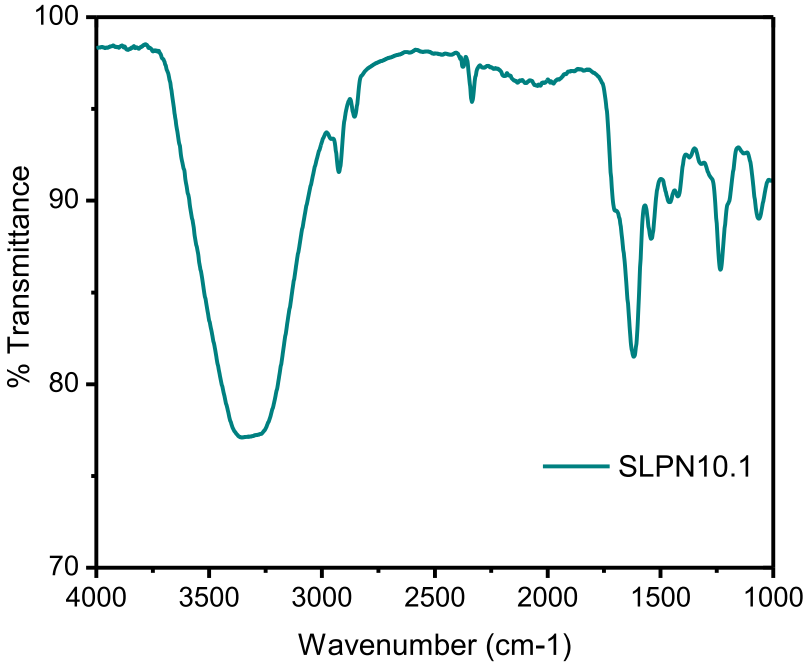

Figure S7. FT-IR spectrum of nanoparticles: SLPN10.1.

Statistical Analysis

The drug release experiments were conducted in triplicate, and the cumulative release (%) data are reported as the mean ± standard deviation (SD) of the three independent measurements. Statistical analysis were performed using two-way ANOVA through Matlab "anova2" function. Results were considered significant at 95% confidence interval ($p < 0.05$). We have conducted analysis for drug release results both at pH 7.4 and at pH 6.5. Both enzyme (*p=0.0001 at pH 7.4, p=0.0001 at pH 6.5*) and time (*p=0.0005 at pH 7.4, p<0.0001 at pH 6.5*) have a statistically significant effect on drug release since $p<0.05$.

References

[1] T. Wang, J. Xue, Q. Hu, M. Zhou, C. Chang, Y. Luo, *Sci. Rep.* **2017**, *7*.
